# Supplementary material for: The interactome of intact mitochondria by cross-linking mass spectrometry provides evidence for coexisting respiratory supercomplexes
Source: Mol Cell Proteomics. 2017 Dec 8;17(2):216–32. doi: 10.1074/mcp.RA117.000470 (PMC5795388; doi:10.1074/mcp.RA117.000470)
Supplement: Supplemental Data [file supp_RA117.000470_133922_0_supp_23978_fzffwf.zip › spectra_annotation/mito_spectra_annotation/343-1-10-1-10-1.pdf]

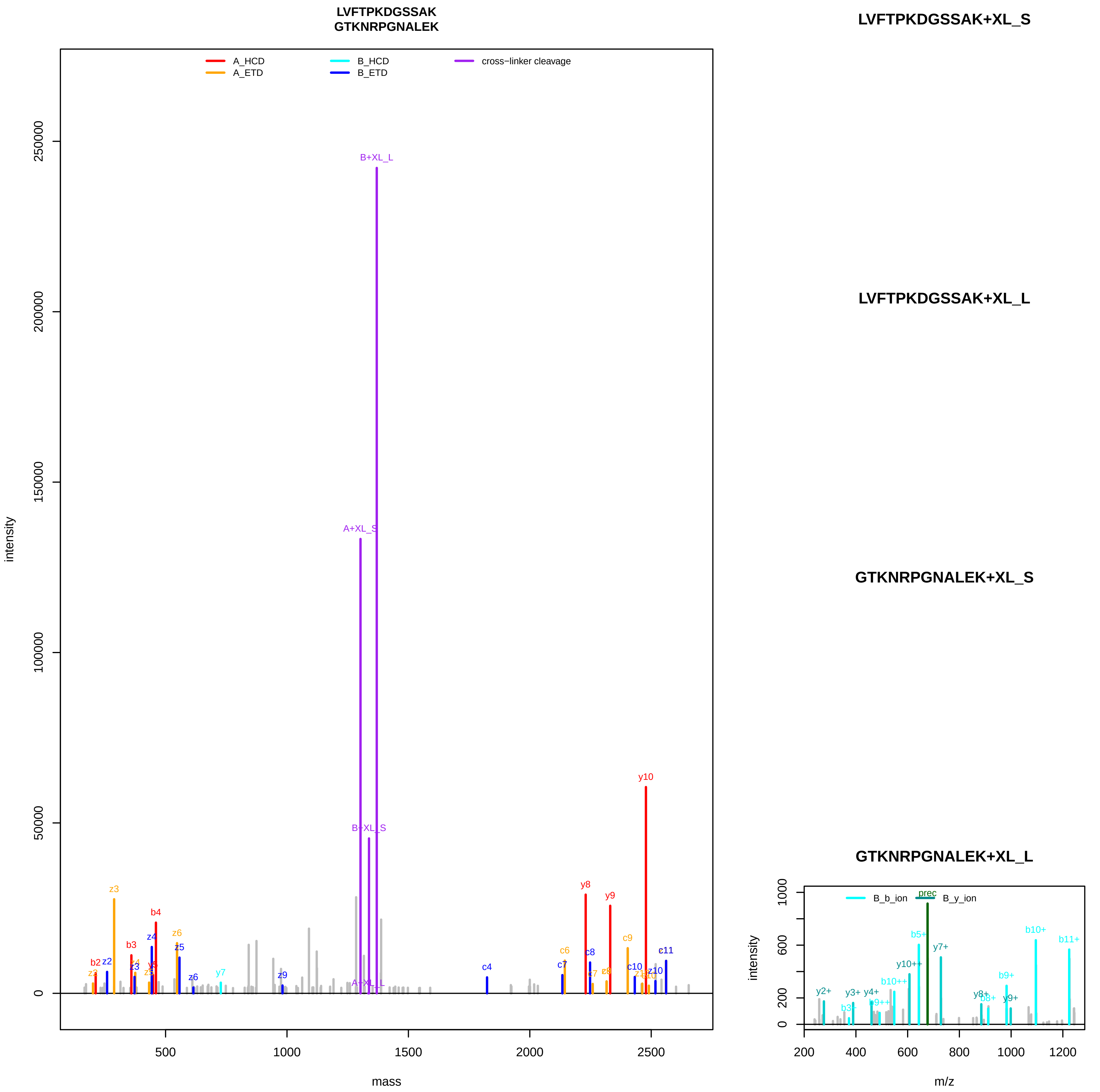

LVFTPKDGSSAK
GTKNRPGNALEK
A_HCD
A_ETD
B_HCD
B_ETD
cross−linker cleavage
B+XL_L
A+XL_S
B+XL_S
A+XL_L
y10
y8
y9
z3
b4
b3
b2
z2
z4
z5
z6
z6
y7
z9
c4
c6
c7
c8
c9
c10
z10
c11
intensity
mass
LVFTPKDGSSAK+XL_S
LVFTPKDGSSAK+XL_L
GTKNRPGNALEK+XL_S
GTKNRPGNALEK+XL_L
B_b_ion
B_y_ion
prec
b5+
y7+
b10+
b11+
y10++
b10++
b9+
y8+
b8+
y9+
y2+
y3+
y4+
b3+
b9++
m/z
